# Eco-evolutionary dynamics between multiple competitors reduce phytoplankton coexistence but have limited impacts on community productivity

**DOI:** 10.1098/rspb.2025.1146

**Published:** 2025-07-09

**Authors:** Charlotte Louise Briddon, Aurora Menéndez García, Giulia Ghedini

**Affiliations:** ^1^ GIMM - Gulbenkian Institute, Lisbon, Portugal; ^2^ Department of Biology, Ghent University, Ghent, Flanders, Belgium; ^3^ School of Biological Sciences, Monash University, Melbourne, Australia

**Keywords:** metabolism, community functioning, biomass, biodiversity, evolution, competition

## Abstract

Species can evolve rapidly in response to competition but how evolution within communities affects community properties is unclear. Niche theory predicts that species should evolve to use different resources, increasing coexistence and community productivity. However, recent experiments suggest that species might instead evolve their competitive ability, particularly when competing for essential resources. To test the consequences of species evolution on community properties, we grew three species of marine phytoplankton in monoculture (alone) or polyculture (together) for 4.5 months. We then combined them in communities based on their competition history and tracked community composition and productivity over time. We found that species dominance was unaffected, but coexistence was reduced when species evolved together (polyculture isolates). These species-level changes did not affect community functions equally. Total biovolume growth rates and carrying capacity were the same between communities of monoculture or polyculture isolates but the latter had greater oxygen fluxes during the exponential phase. Our results suggest that evolution within communities can strengthen competitive differences between species with uneven effects on community functioning. While some community properties seem robust to species evolutionary changes, we should be cautious in extrapolating the consequences of evolution from community biomass to other aspects of productivity or stability.

## 1. Introduction

Ecological interactions can result in rapid evolution. While there is increasing evidence of the effects of eco-evolutionary dynamics on the traits of organisms [1,2], the consequences for community properties remain unclear, particularly for eukaryotes. In the presence of competitors, organisms often evolve at different rates or in different directions than organisms alone [3,4]. Understanding these evolutionary trajectories and how they affect community properties is important to forecast the consequences of biodiversity changes, which are altering community composition and increasing the probability that species will encounter new competitors.

Niche theory predicts that species which compete for similar resources should evolve distinct phenotypes when they coexist in the same areas (sympatry) compared to when they exist in isolation [5–7]. Such character divergence can facilitate coexistence by promoting niche partitioning [8–10]. Patterns of niche differentiation are sometimes observed in studies of experimental evolution on bacteria, where species can evolve to preferentially use the waste products of other species, thus increasing facilitative interactions [4] (although this is not always the case [11]). Over generations, trait divergence can improve species fitness and increase community productivity owing to more efficient resource use [4,12,13].

In species that compete for essential resources, such as plants or algae, the evolution of niche partitioning might however be rare [14]. In these cases, competition might be more frequently selected for competitive ability rather than the type of resources used [15]. Changes in competitive ability can also have important consequences for community structure and functioning. For instance, changes in competitive ability might facilitate coexistence if they occur in parallel across species because they can reduce fitness differences [1]. Improvements in competitive ability can also enhance the overall efficiency of resource use [16,17], increasing community productivity and potentially constraining the establishment of invaders [18]. However, beyond bacteria [4,10], tests for how evolutionary processes between multiple competitors affect community properties are rare [19,20]. The scarcity of tests leaves us blind to the potential changes in community functioning that can ensue from rapid species evolution.

**Figure 1.**
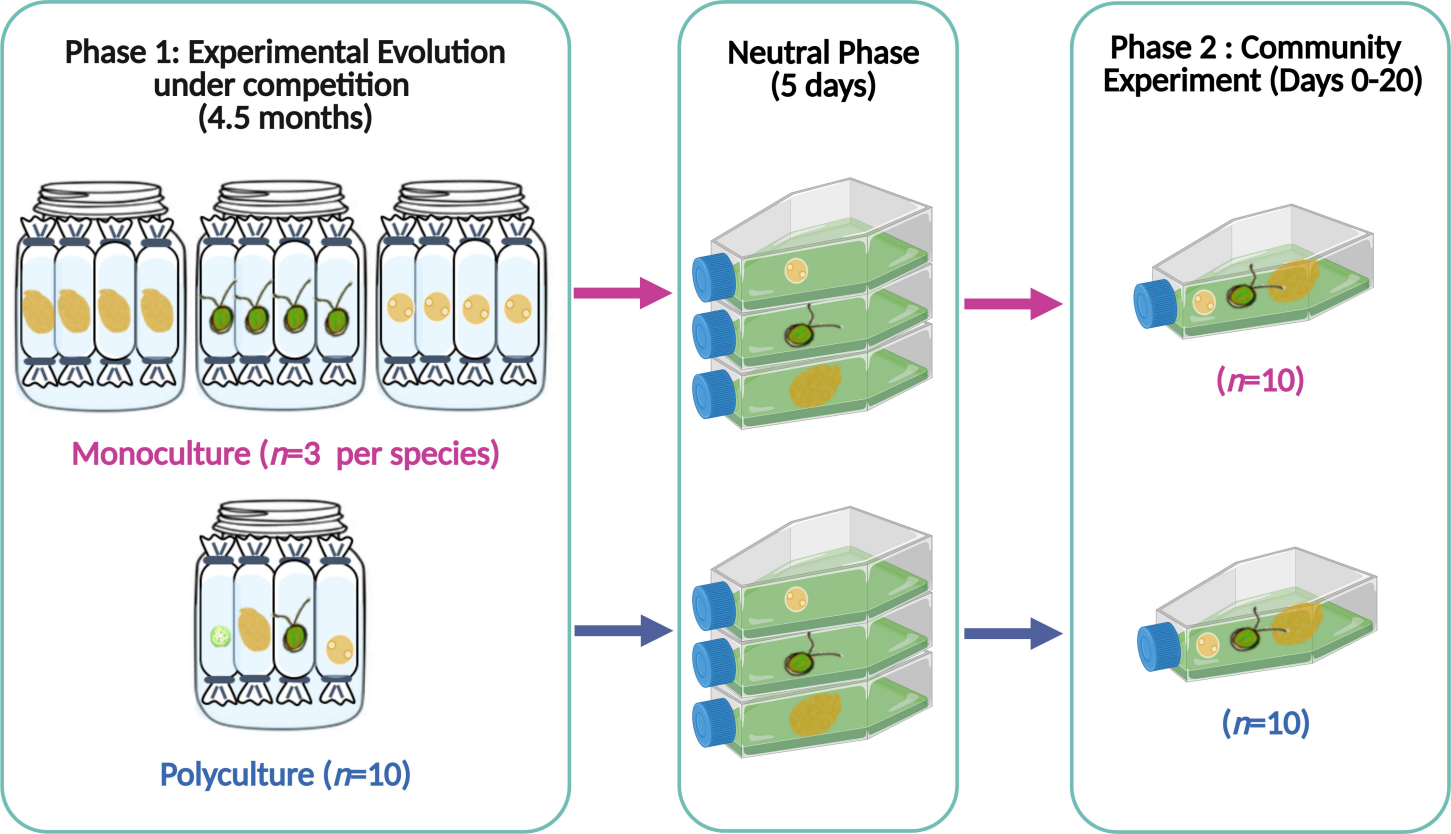
Schematic showing the different experimental phases. During phase 1 (experimental evolution), we evolved three phytoplankton species (*Amphidinium*, *Dunaliella*, *Tisochrysis*) under either intraspecific (monoculture) or interspecific competition (polyculture) for 4.5 months, using dialysis bags that allow competition but keep these species separate (the fourth species, *Nannochloropsis*, was competitively excluded halfway during the evolution phase and thus was not included in the following experiments). After 4.5 months, we combined the species with a shared competition history for phase 2 (community experiment; days 0−20). Our goal was to assess how competition history impacted community assembly and functioning. Before phase 2, we grew each species in an individual flask for 5 days to remove any plastic response (neutral phase). Created in BioRender. https://biorender.com/d74z269

Phytoplankton provides a useful model system to study evolution in communities of eukaryotic organisms because phytoplankton has rapid cell division (approx. 1 per day when resources are not limited [21,22]), large population sizes and therefore high capacity for evolution [23,24]. These organisms perform important ecosystem functions because they transfer energy to foodwebs and drive the carbon cycle [25]. So, understanding how eco-evolutionary processes influence the functioning of phytoplankton communities is important. However, maintaining multiple species in the same environment for enough time to allow evolution is difficult in the laboratory [20]. To solve some of these difficulties, we used an experimental approach based on the use of dialysis bags which is a close but imperfect representation of evolution in a community. Specifically, we experimentally evolved three phytoplankton species together in a community (polyculture) for 4.5 months by enclosing each species in a separate ‘cage’ (dialysis bag)—so that species competed for nutrients but were physically separated (figure 1). We did the same thing for species alone to simulate intraspecific competition (enclosing replicate populations of the same species in dialysis bags). Dialysis bags have been used in many experiments before to expose organisms to the same environmental conditions while keeping them physically separated [6,26–29]. After 17 weeks of evolution, each species was isolated to quantify how their traits evolved in common garden experiments; this work showed that all species increased their competitive ability when tested alone. Specifically, each species reduced its sensitivity to intraspecific competition (lowering the density-dependence of growth and energy use), but these responses were weaker for species that evolved in polyculture [30].

Our goal here is to determine the community effects of these evolved changes and whether they differ for communities of species evolved alone (monoculture isolates) or together (polyculture isolates). We hypothesize that (i) communities of polyculture isolates should show greater coexistence than communities of monoculture isolates because species had the opportunity to adapt to each other. While phytoplankton compete for essential resources, these species can partition some resources such as light [31,32] so evolution with interspecific competitors might result in (some degree of) niche differentiation. Furthermore, all species reduced their sensitivity to intraspecific competition [30], thus potentially equalizing fitness differences. We also predict that (ii) communities of polyculture isolates should be more productive because resources should be more fully utilized by the community members (either through niche partitioning or increased resource use efficiency).

## 2. Material and methods

To test the consequences of evolution with competitors on community assembly and functioning, we used three species (strains) of marine phytoplankton (*Amphidinium carterae* RCC88*, Dunaliella tertiolecta* RCC6 and *Tisochrysis lutea* RCC90) acquired from the Roscoff Culture Collection, France. These strains have been maintained in the Roscoff Culture Collection for years, so they have not experienced interspecific competition for many generations, increasing the likelihood of observing adaptation between them. The strains were not clonal and belong to different and widely distributed taxonomic algal groups: they have different morphologies and cell sizes, encompassing diverse competitive and physiological traits (*Amphidinium;* initial average cell size monoculture isolates = 779.53 ± 32.14 µm^3^; polyculture isolates = 773.07 ± 16.29 µm^3^; *Dunaliella* = 387.03 ± 28.52 µm^3^; 375.86 ± 15.13 µm^3^; *Tisochrysis =* 71.53 ± 3.34 µm^3^; 70.46 ± 1.69 µm^3^).

First, we evolved these species either alone (‘monoculture’) or together (‘polyculture’) for 4.5 months (§2a; details below). The evolution phase also included a fourth species, *Nannochloropsis granulata* (19 ± 0.17 µm^3^; RCC438), from the same phytoplankton collection. However, *Nannochloropsis* was competitively excluded in the polyculture treatment after 16 weeks, therefore it was not included in subsequent experiments. After phase 1, we combined the evolved species in communities based on their competition history to determine how communities of species evolved together (polyculture isolates) perform relative to novel communities (monoculture isolates) (§2b).

### (a) Phase 1: experimental evolution

To evolve each species in monoculture or polyculture we used dialysis bags (MWCO 14 kDa, pore size 25 Angstrom, Dialysis membrane Membra-CEL, Carl Roth, Germany), which allowed competition for nutrients and exchange of metabolites but maintained a physical separation between species [26,27,33]. This approach was necessary to: (i) characterize the traits of each evolved species in isolation (after 17 weeks of evolution) [30]; and (ii) establish and compare communities of monoculture and polyculture isolates with the same initial composition (after 19 weeks of evolution—this study).

For the intraspecific treatment (monoculture), we established three replicate beakers per species. Each beaker contained four dialysis bags, each filled with the same species surrounded by enriched seawater medium (f/2 media prepared from 0.2 µm filtered and autoclaved natural seawater [34]) containing no phytoplankton. For the interspecific treatment (polyculture), we set up 10 replicate beakers using the same design but each of the four dialysis bags within a beaker was filled with a different species (figure 1). Each dialysis bag had the same initial biovolume of 5.2 × 10^9^ μm^3^, filled to a volume of 45 ml with f/2 media (biovolume density = 1.16 × 10^5^ μm^3^ μl^−1^; for reference the carrying capacity of species alone is approx. 10^6^ μm^3^ μl^−1^). Once a week, we transferred a set volume of 25 ml from each dialysis bag to a new sterilized bag and beaker. The contents of the bag were topped up with 20 ml of fresh media to a constant total volume of 45 ml. At each transfer, we also replaced the media in the beaker. We kept the dilution factor low to maintain species under conditions of intense competition throughout the evolution phase. For weeks 5−10, we increased the dilution factor, transferring 15 ml from each bag (instead of 25 ml); this change was necessary to increase the proportion of resources available and keep populations growing. Importantly, this was done across all treatments and replicates. Light intensity was set at 60 µmol m^−2^ s^−1^ with a 12 L : 12 D schedule using low-heat 50 W LED floodlights (Surface Luminária LED 230V, Robert Mauser, Portugal). The temperature was maintained at 20 ± 1℃. Further details of this experiment are described in [30].

### (b) Phase 2: community experiment

After 4.5 months of evolution (19 weeks), we collected 10 samples (20 ml) for each species and competition treatment (monoculture and polyculture, *n* = 60) and placed them alone in a neutral environment (cell culture flasks filled up to 100 ml with f/2 media) for 5 days to remove any environmental conditioning. For the monoculture treatment, we sampled multiple bags (populations) from the same beaker (since we had 12 bags in total divided between three beakers per species). We recognize this is not ideal because these populations are not independent replicates as they share the same beaker. However, there was no ‘beaker’ effect on the traits of the individual populations [30]; furthermore, the populations evolved separately within each bag for 4.5 months, so they do not share genetic mutations that appeared during this time.

After the neutral phase, we used the populations in the individual flasks to assemble communities of novel species (by randomly combining monoculture isolates of the three species) and communities of species that evolved together (by combining polyculture isolates, in this case, we combined species that came from the same beaker; *n* = 10 for each community type). All communities were started with an equal biovolume of each species (1.06 × 10^4^ µm^3^ µl^−1^) which was added to 250 ml culture flasks, filled up to 100 ml with f/2 media (figure 1). Phase 2 lasted for 20 days, until the samples reached carrying capacity. Each sampling day, we collected 10 ml from each culture flask for analysis (see details below) and replaced it with 10 ml of fresh media. The sampling took place on days 2, 4, 6, 8, 10, 12, 14, 17 and 20.

### (c) Cell size, densities and biovolume

On each sampling day during phase 2, we measured cell size (µm^3^), cell circularity, cell density (cells µl^−1^) and biovolume (µm^3^ µl^−1^; calculated by multiplying cell density and average cell size for each replicate). Briefly, we fixed 1 ml of sample from each replicate with 1% Lugol and acquired 20 photos with an inverted Olympus microscope (400 x magnification). The size (length and width) and abundance of each species were recorded using ImageJ and Fiji software (version 2.0) [35]. We then estimated cell volume by assigning either a prolate spheroid shape (*Amphidinium*, *Dunaliella*) or spherical shape (*Tisochrysis*) to each cell.

### (d) Metabolic rates

In conjunction with biovolume data, we measured photosynthesis, post-illumination and dark respiration rates to determine changes in community metabolism. For each community, we collected 5 ml and placed it in a vial with an integrated oxygen sensor. Vials were placed on 24-channel optical fluorescence oxygen readers to measure oxygen production and consumption (PreSens Sensor Dish Reader, SDR; PreSens Precision Sensing, Germany) [26,30]. The samples were measured for 20 min in the light to quantify photosynthesis (the first 3 min of measurements were discarded to allow acclimation), followed by 40 min of darkness to determine respiration rates (the first 15 min were used to quantify the post-illumination rates and the remainder the dark respiration rates). As the cultures were not axenic, eight blanks per day were filled with spent media with no phytoplankton cells to account for the background bacterial activity (spent media was obtained by centrifuging samples at 5000 rpm for 10 min to separate the algal cells from the supernatant).

The photosynthesis and respiration rates (VO_2_; μmol O_2_ min^−1^) of each sample were calculated as:


$${VO}_{2}=\frac{m_{a}-m_{b}}{100}\;\times \;V\beta O_{2},$$

where *m*_a_ is the rate of change in O_2_ levels of the sample (min^−1^), *m*_b_ is the mean O_2_ level across all blank samples (min^−1^), *V* is the sample volume (0.005 l) and *β*O_2_ is the O_2_ capacity of air-saturated seawater at 20℃ and 35 ppt salinity (225 µmol O_2_ l^−1^) [36]. Subsequently, we converted the photosynthetic and respiration rates (µmol O_2_ min^−1^) to calorific energy (J min^−1^), using a conversion factor of 0.512 J µmol O_2_^−1^ to estimate the energy production and consumption, respectively [37]. These calculations were completed using the LoLinR package [38] in R (version 4.3.2) [39].

### (e) Cell DNA content and pigment fluorescence

To explore whether differences between monoculture and polyculture isolates could stem from differences in cell traits, we quantified the amount of DNA (nucleus volume) relative to cell size. Cells with smaller genomes (relative to their size) should have higher fitness because DNA is energetically costly, and redundant DNA should be under selection to be eliminated in highly competitive (resource poor) environments [40,41]. Previous work on phytoplankton only partially supports these predictions: cells with relatively less DNA for their size have higher maximum growth rate and total biovolume but lower energy fluxes [42]. Therefore, concomitantly to DNA content and biovolume, we quantified total pigment fluorescence as an indication of the amount of photosynthesis (energy) cells can sustain. Preliminary tests showed that we could not measure DNA content in *Tisochrysis* because of its small size, so we collected these data on the two larger species (*Amphidinium* and *Dunaliella*).

We used 4’,6-Diamidine-2’-phenylindole (DAPI) staining to quantify nucleus volume as a proxy of DNA content [42]. This analysis was carried out on individual populations isolated at the end of phase 1. We used six random populations (bags) for each species and competition treatment. The DAPI did not bind well to some samples, so we processed four monoculture and five polyculture populations for *Amphidinium*, and five monoculture and six polyculture populations for *Dunaliella*. Samples of each population were standardized to 3 × 10^6^ cells ml^−1^. Then, 1 ml samples were fixed with 2% glutaraldehyde, washed and resuspended in growth medium, then stained with 0.1 µg ml^−1^ DAPI (4′,6-diamidino-2-phenylindole) and stored in the dark for 30 min (as described in [42]). DAPI can penetrate dead cells and bind with the DNA to form a fluorescent stain which can be measured with fluorescence microscopy. After the dark incubation, the samples were imaged using the ZEISS Axio Imager with Apotome 2 microscope, using the DAPI channel (excitation: 385 nm) to measure the nucleus size and brightfield view for the cell size. For each population, we took 50 photographs with both the brightfield view and the DAPI channel. We analysed the photographs using ImageJ and Fiji software (version 2.0) to estimate the nucleus and cell size (assuming spheroid shapes for both) of at least 120 cells for each species (total cell measured: 189 mono, 134 poly for *Amphidinium*; 120 mono, 150 poly *Dunaliella*). Previous work showed that organelle DNA contributed very little to overall variation in phytoplankton DNA content [42], so we used the nucleus volume of a cell as a proxy for its DNA content.

Concomitantly, we used spectral flow cytometry to estimate the per cell total mean pigment fluorescence of the same two species (*Amphidinium* and *Dunaliella*). We used pigment fluorescence as a proxy of photosynthetic potential which should be less plastic than photosynthesis or respiration rates (and also because we could not measure photosynthesis or respiration rates of individual species directly in the community but could only measure the total). Samples were analysed using the Cytek Aurora (Cytek Biosciences, USA) equipped with four lasers emitting at violet (405 nm–100 mW), blue (488 nm–50 mW), yellow green (561 nm–50 mW) and red (640 nm–80 mW). A control of enriched seawater medium (f/2 media) was used to define experiment settings and the gating strategy, allowing for the removal of background noise (electronic supplementary material, figures S1, S2). The signal threshold was set on the forward scatter at 30 000. The unique spectral fluorescence profile of each species was considered as an individual reference control for spectral unmixing. After acquiring both populations of *Amphidinium* and *Dunaliella* separately as different reference controls, unmixing with autofluorescence extraction was performed and samples were acquired. For each sample, we collected at least 10 000 events (up to a max. of 20 000 events) using the previously established gating strategy and the unmixed fluorescent parameters created for each phytoplankton species (electronic supplementary material, figures S1, S2), allowing us to extract the per cell total mean pigment fluorescence. We analysed four monoculture populations for each species, six polyculture populations for *Amphidinium* and ten for *Dunaliella*, using the SpectroFlo software (version 3.3.0, Cytek Biosciences). The different number of populations was owing to the low cell density of some populations which made them unsuitable for this analysis.

### (f) Data analysis

The statistical analyses were done on the data collected during phase 2 (community experiment), except for the DNA content and pigment fluorescence analyses which were done on individual populations collected from phase 1. All data were analysed using R (version 4.3.2) [39] and Rstudio [43] with the packages nlme [44], lme4 [45], emmeans [46], car [47], plyr [48], vegan [49] for analyses and ggplot2 [50,51]. For all statistical analyses, we decided whether to keep or remove non-significant interactions between the variables by computing the Akaike information criterion (AIC) for the model with and without the interaction, and then selecting the model with the lowest AIC. All figures with means show the least square means with a 95% confidence interval.

#### (i) Differences in species growth dynamics

Using data from phase 2, we tested how each species performed in the community based on its competition history (monoculture, polyculture). Using biovolume data (μm^3^ μl^−1^), we calculated the maximum rate of increase (*r*_max_) and the maximum value (i.e. carrying capacity, *K*) for each species within each community, similarly to [30]. Briefly, we fitted four models to biovolume data: a logistic-type sinusoidal growth model with lower asymptote forced to 0, a logistic-type sinusoidal growth model with non-zero lower asymptote, a Gompertz-type sinusoidal growth model and a modified Gompertz-type sinusoidal growth model including population decline after reaching a maximum. Then we used AIC to determine the best-fitting model for each population with successful convergence. From this model, we estimated the maximum predicted value (*K*) of biovolume for each species in each community. From the first derivative, we extracted the maximum rate of increase (*r*_max_). We then tested for differences in these parameters (*r*_max_ and *K*) of each species separately using a linear model that included initial biovolume as covariate and competition history (monoculture and polyculture isolates) as a factor, including their interaction. We performed this same analysis on the cell densities for each species (cells μl^−1^).

To visualize differences in the growth trajectory of each species between the two community types, we used the same models above, but we fitted them across all replicates of each species within a treatment. We then plotted the best-fitting model based on AIC.

#### (ii) Changes in cell size

For each species (phase 2), we tested for differences in cell size and shape owing to their competition history. We used a linear mixed-effect model that included competition history and experiment day as factors (with their interactions), with sample code as a random effect to account for repeated measures.

#### (iii) Evenness

We calculated Pielou’s evenness index (*J*’) based on the biovolume of the individual species to estimate changes in community composition (including species diversity and relative abundances) [52]:


$$J^{\prime }=\frac{H^{\prime }}{ln\left(S\right)},$$

where *H*’ is the Shannon–Weiner diversity index and *S* is the number of species in the sample. To determine if species evenness differed owing to competition history, we used a linear mixed-effect model with evenness as a response variable, competition history and experiment day as factors (including their interaction), and sample code as a random effect.

#### (iv) Differences in community biovolume

We used the same models described above in §2a but applied to the total community biovolume to estimate *r* and *K*, fitting these models to each community replicate. We then tested differences in *r* and *K* (separately) between monoculture and polyculture communities using a linear model, including initial biovolume as a covariate. We refitted these models across all replicates within each competition treatment to show changes in community biovolume over the entire experiment.

#### (v) Community metabolic rates

We tested how competition history affected the relationship between community oxygen rates and total biovolume (phase 2), running this analysis for photosynthesis, post-illumination, and dark respiration rates separately. We also estimated daily net energy production as 12 h of energy produced through photosynthesis minus 12 h of respiration (calculated as 15 min of post-illumination and 11.75 h of dark respiration). We excluded data from day 17 in the analysis of post-illumination rates (and thus also in the analysis of net energy production) because all samples had unusually low post-illumination rates on that day that did not fit with the trajectory observed over time.

We analysed the oxygen rates separately for the exponential (until day 10 included based on community biovolume trajectories) and stationary phase (until day 20) because the relationship between oxygen rates and biovolume is different between these two phases (a positive relationship in exponential phase versus no relationship in stationary phase). Within each growth stage, we used linear mixed-effect models with the metabolic rates as a response variable, biovolume and competition history as predictors (including their interaction), and community sample code as a random effect. Since variances were heterogeneous for the exponential phase of photosynthesis, post-illumination respiration and net energy production, we used a generalized least squares model to apply a treatment-specific variance to each competition treatment.

#### (vi) DNA content

To determine if competition treatment influenced the amount of DNA, we used a linear mixed-effect model with nucleus volume as a response variable, cell volume and competition treatment as predictors (including their interaction), and dialysis bag nested in beaker as random effect for each species separately. Data of nucleus and cell volume from *Amphidinium* were log_10_-tranformed prior to analyses to meet normality assumptions. We only analysed cells of comparable sizes between the two competition treatments, so we excluded cells greater than 2400 and 500 μm^3^ for *Amphidinium* and *Dunaliella,* respectively, which were only present in the monoculture populations (the pattern between monoculture and polyculture treatment was the same with or without these cells; electronic supplementary material, figure S7).

#### (vii) Total pigment fluorescence

To determine the influence of competition treatment on pigment content, we used a linear model (for each species individually) with the total pigment fluorescence (mean per cell) as the response variable and competition treatment as a factor. Because of the fewer data available (one point per population), we used a simple linear model instead of a mixed model. We did not include cell size as a covariate because the average pigment fluorescence per cell is calculated over many cells (>10 000 events per sample) and there were no significant differences in size between monoculture and polyculture populations of each species (electronic supplementary material, figure S6).

## 3. Results

### (a) Competition history maintains species dominance but reduces evenness

**Figure 2.**
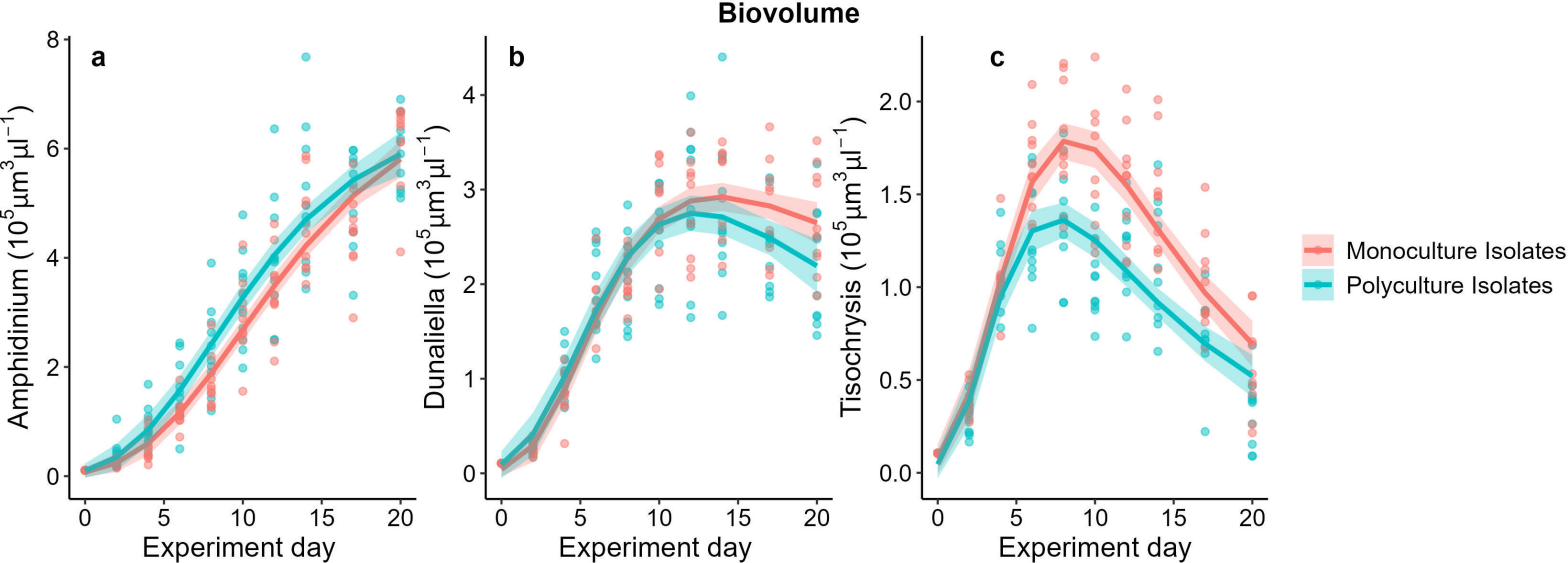
Monoculture and polyculture isolates of each species show similar biovolume growth dynamics initially (see the electronic supplementary material, figure S3 for *r* and *K* estimates), but differences in species dominance become more marked in polyculture isolates as competition increases. *Amphidinium* dominated in terms of biovolume in both treatments (a), whereas *Dunaliella* (b) and *Tisochrysis* (c) declined from days 14 and 8, respectively—and more so in communities of polyculture isolates.

**Figure 3.**
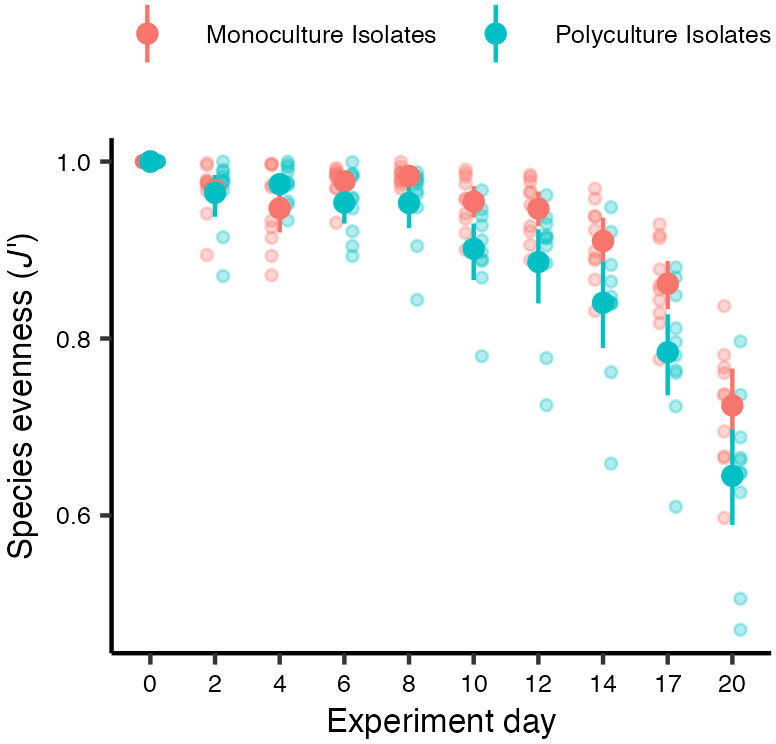
The evenness of polyculture isolates (species evolved together) declined faster over time compared to that of monoculture isolates (species evolved alone). Refer to the electronic supplementary material, table S2 for post-hoc tests for each day.

Competition history initially did not affect how species performed within their community: monoculture and polyculture isolates of each species had similar maximum growth rates (*r*_max_) (*Amphidinium*: *F*_1,17_ = 1.44, *p* = 0.25; *Dunaliella*: *F*_1,17_ = 0.65, *p* = 0.43; *Tisochrysis*: *F*_1,17_ = 3.36, *p* = 0.08; electronic supplementary material, figure S3, table S1). As biovolume increased, however, the difference between dominant (*Amphidinium*) and subordinate species (*Dunaliella*, *Tisochrysis*) started to appear and more so in polyculture isolates (figure 2). Polyculture isolates of *Tisochrysis* reached a lower carrying capacity (*F*_1,17_ = 14.12, *p* = 0.0016), while there was no significant difference for *Dunaliella* (*F*_1,16_ = 2.34, *p* = 0.15) or *Amphidinium* (*F*_1,17_ = 1.37, *p* = 0.26; electronic supplementary material, figure S3, table S1). The two weaker competitors (*Dunaliella* and *Tisochrysis*) experienced mortality (a decline in biovolume) soon after reaching their maximum biovolume, after 14 and 8 days, respectively, while the biovolume of the dominant species *Amphidinium* remained on an upward trajectory. This pattern was observed in both community types but was stronger for species that evolved in polyculture (figure 2). Consequently, evenness declined faster in communities of polyculture isolates (species evolved together) compared to monoculture isolates (species evolved alone; competition history × experiment day: *F*_9,162_ = 4.23, *p* < 0.0001; figure 3; electronic supplementary material, table S2). The dynamics of species biovolume mirrored those of cell densities (electronic supplementary material, figures S4, S5, table S3) because competition history did not consistently affect cell size or circularity for any of the species (electronic supplementary material, figure S6, table S4).

### (b) Small differences in cell traits might influence coexistence dynamics

**Figure 4.**
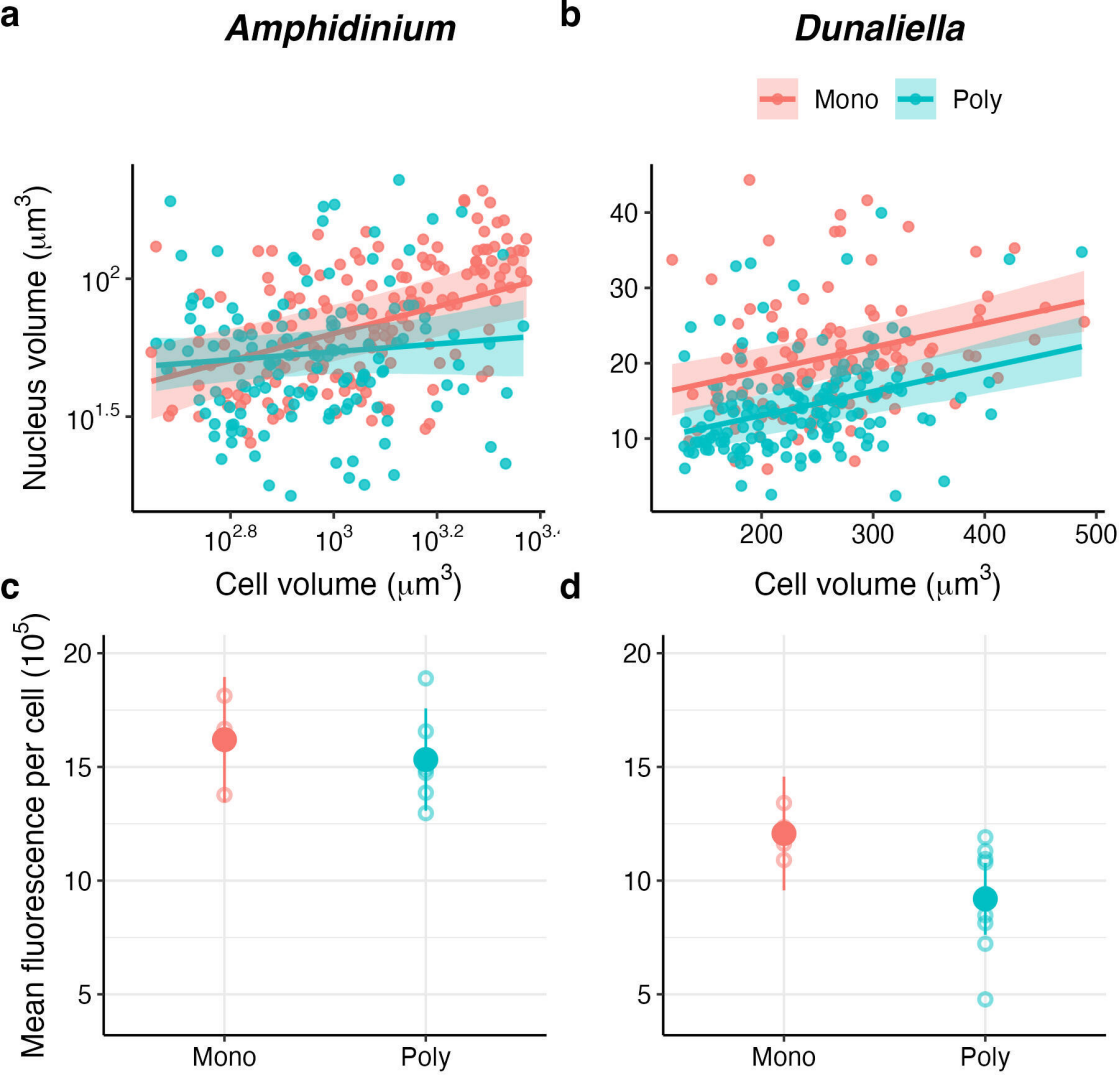
Polyculture-evolved populations of both *Amphidinium* and *Dunaliella* had lower DNA content relative to their cell size (a,b). This reduction in DNA content did not increase fitness (as observed in figure 2; electronic supplementary material, figures S3, S5). On the contrary, *Dunaliella* populations evolved in polyculture had a significantly lower total pigment fluorescence (mean per cell; d), suggesting a lower capacity for energy production which could explain its lower performance in the community. The difference between polyculture and monoculture-evolved populations was not significant for *Amphidinium* (c).

To determine what could drive these differences in performance and coexistence between species evolved in monoculture or polyculture, we quantified the amount of DNA content (nucleus volume) and pigment fluorescence per cell for *Dunaliella* and *Amphidinium* (we could not quantify DNA in *Tisochrisys* because of its smaller cell size). For both species, cells evolved in polyculture had lower DNA content relative to their size (figure 4a,b) as could be expected for cells evolved under intense competition—this pattern was visible only in larger cells for *Amphidinium* (log_10_(cell size) × competition history: *F*_1,246_ = 4.60, *p* = 0.03) and across all range of cell sizes for *Dunaliella* (competition history: *F*_1,5_ = 7.31, *p* = 0.04; electronic supplementary material, table S5). The reduction in DNA content was not associated with an increase in fitness since *r* and *K* were similar between monoculture and polyculture isolates for both species (electronic supplementary material, figures S3, S5). On the contrary, the reduction in DNA content in polyculture isolates was associated with a lower mean pigment content for *Dunaliella* (*F*_1,12_ = 6.16, *p* = 0.03) but not for *Amphidinium* (*F*_1,8_ = 0.45, *p* = 0.52; figure 4c,d; electronic supplementary material, table S6), possibly contributing to their different performance in the community as competition increased (polyculture-evolved *Dunaliella* performed worse than monoculture-evolved *Dunaliella* in competition with other species, while *Amphidinium* performed similarly; figure 3).

### (c) Community biovolume and oxygen production respond differently to competition history

**Figure 5.**
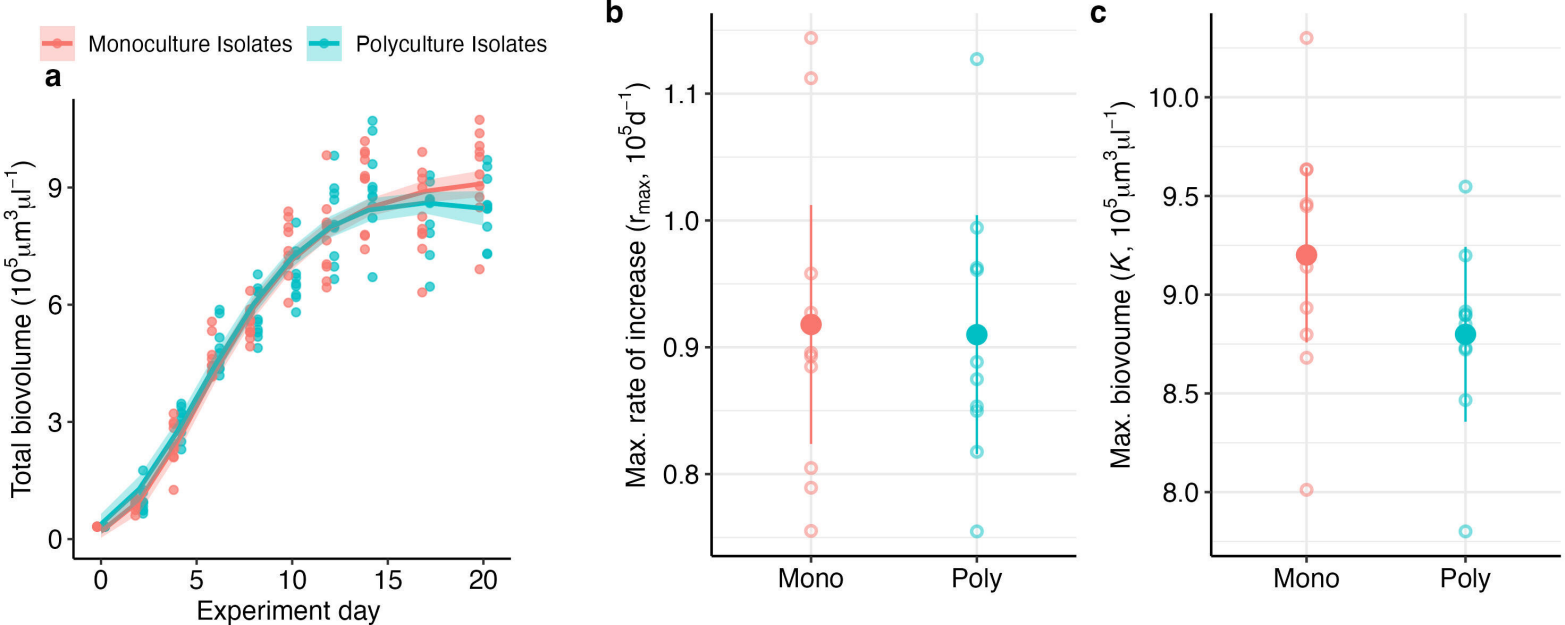
Total biovolume (μm^3^ μl^−1^) increased identically over time in communities of polyculture and monoculture isolates, up until carrying capacity (approx. day 20) (a). There were no significant differences in the maximum rate of biovolume increase (b) or carrying capacity (c).

Despite the differences in species evenness and traits, total biovolume increased identically in the two community types (figure 5a). Monoculture and polyculture isolates had the same maximum rates of increase and maximum values of total biovolume by day 20 (figure 5b,c; electronic supplementary material, table S7). However, the two community treatments had different oxygen evolution rates, albeit these differences were weak and mostly visible during the exponential phase.

**Figure 6.**
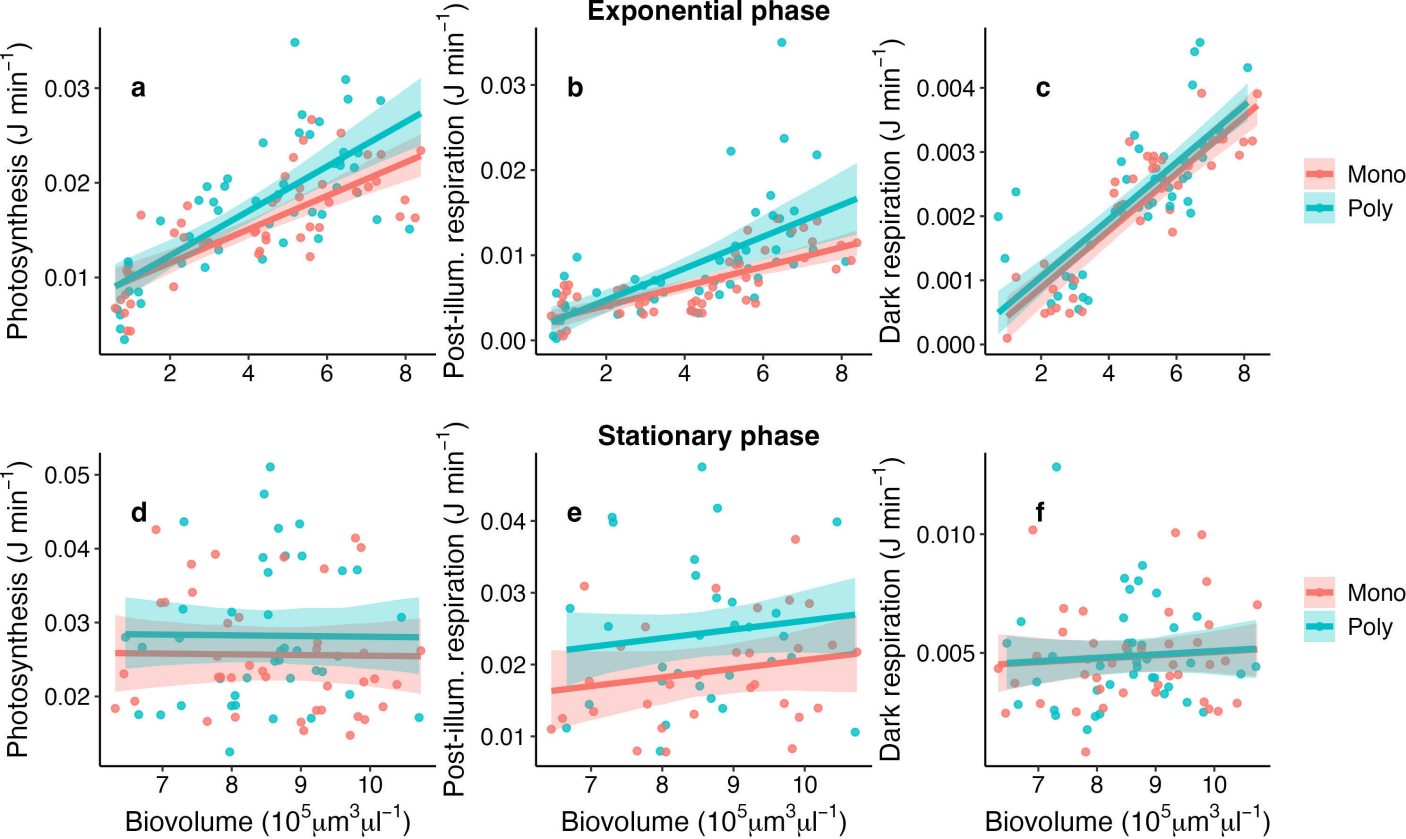
Differences in community oxygen rates as a function of total biovolume: photosynthesis (a,b), post-illumination (c,d) dark respiration (e,f), and net daily energy production (g,h). Panels on the left refer to the exponential growth phase (days 0−10), panels on the right to the stationary phase (days 12−20). Refer to the electronic supplementary material, table S8 for the model outputs.

In exponential phase, the polyculture isolates tended to have higher photosynthesis (borderline competition history effect: *F*_1,18_ = 4.32, *p* = 0.0533; electronic supplementary material, table S8) and post-illumination rates (competition history effect: *F*_1,18_ = 7.15, *p* = 0.016; borderline biovolume × competition history effect: *F*_1,77_ = 3.94, *p* = 0.0508) compared to the monoculture isolates (figure 6). In the stationary phase, post-illumination rates were still significantly higher for the polyculture isolates (competition history: *F*_1,57_ = 5.6, *p* = 0.03), while photosynthesis rates were similar between treatments (*F*_1,18_ = 0.98, *p* = 0.34). Dark respiration rates were not affected by competition history, either in the exponential or in the stationary phase (figure 6; electronic supplementary material, table S8). Therefore, when we estimated net energy production over 24 hours, we found the same pattern observed for photosynthesis: net energy production was marginally higher in communities of polyculture isolates during the exponential phase (competition history effect: *F*_1,18_ = 4.37, *p* = 0.0496; electronic supplementary material, figure S8, table S8) but not during the stationary phase (*F*_1,18_ = 1.92, *p* = 0.18).

## 4. Discussion

When species co-occur with competitors, they can evolve to increase niche differences and/or competitive ability. While niche theory anticipates that increased niche differences should be the most likely outcome, empirical tests more consistently show changes in competitive ability, particularly when species compete for essential resources [1,18,53]. However, the consequences of these evolved changes for community properties are unclear. To answer this question, we investigated how evolution with multiple competitors influenced phytoplankton coexistence and the productivity of their communities in terms of two important functions (biomass production and oxygen fluxes).

Our results suggest that, even if niche partitioning is possible in phytoplankton [32], evolving with multiple competitors does not facilitate coexistence in this system, at least for the species we studied. On the contrary, evolution with competitors weakened coexistence by strengthening competitive differences—without altering species hierarchies. Indeed, communities of monoculture and polyculture isolates assembled in similar ways and were dominated by the same species (*Amphidinium*), but dominance patterns were stronger in polyculture isolates. These consistent patterns might arise because competition selects for competitive ability rather than niche differences [30] and because species might improve their competitive ability for multiple resources in parallel [54,55].

Recent work shows that all three species included in the communities (*Amphidinium*, *Dunaliella*, *Tisochrysis*) reduced their sensitivity to intraspecific competition after evolution when tested alone, independently of whether they evolved in monoculture or polyculture [30]. The similarity of this response between species was not sufficient to reduce their fitness differences and improve coexistence; instead, a single species (*Amphidinium*) dominated in all communities. We think it is because *Amphidinium* showed stronger evolutionary changes in comparison to the other two species, particularly in terms of energy fluxes (its cells produced higher amounts of net energy in dense populations) [30], possibly explaining its dominance in the community. *Amphidinium* is also a mixotroph [56] so, when competition is intense, this species might increase energy gains by complementing photosynthesis with heterotrophy [57]. Whatever the specific mechanism, higher rates of energy production when resources are scarce might have favoured *Amphidinium* at the expense of the other species (*Tisochrysis*, *Dunaliella*).

If all species evolved in the same direction, why is coexistence reduced in polyculture isolates? The fact that all three species reduced density-dependence in response to intra- or inter-specific competition suggests no trade-offs between evolving alone or with competitors—it actually suggests that reducing density-dependence can be a way to cope with both intra- and inter-specific competition [15,30]. Therefore, the reduced coexistence observed in polyculture isolates is somewhat surprising. However, small differences in density-dependence might become important in the presence of interspecific competitors and trade-offs in competitive ability might be hidden in other traits [58]. For instance, reductions in density-dependence were weaker for species evolved in polyculture [30] possibly because of smaller population sizes or multiple, concomitant selective pressure [6,59]. Furthermore, evolving with interspecific competitors affected two cell traits related to fitness: we observed declines in cell DNA and pigment content, at least for some species evolved in polyculture (*Dunaliella*), which could indicate the loss of important functions and reduction in the capacity for energy production. Highly competitive environments (such as communities) are expected to select for cells with less DNA (since DNA is costly) [42]; but contrary to what the ‘selfish DNA hypothesis’ predicts, these reductions in DNA did not benefit polyculture-evolved cells, neither alone [30] nor in competition with other species. If anything, we observed the opposite, and it seemed that performance was more affected when species were in communities. These results attest to the complex relationship between DNA content and fitness, which remains unclear possibly because of the contrasting effects of DNA on population parameters and energy fluxes [42]. Altogether, small differences in density-dependence and energy use had minor effects on performance when species were alone [30] but became more important as species dynamics unfolded in communities. These data point to the existence of ‘hidden’ trade-offs between competitive ability alone versus in the presence of other species [53], which could explain why evenness declined faster in communities of polyculture isolates.

The reduced coexistence between (co)evolved species contrasts with predictions of character displacement [60,61] that however seem more likely when species compete for substitutable resources. Experiments in bacteria show that character displacement occurs [13,62] and that bacteria often develop facilitative interactions via cross-feeding [4]. These types of interactions however might be less common in other organisms, particularly when resources cannot be easily substituted [63]. A limitation of our work is that, during the experimental evolution phase, species competed for nutrients but were physically separated so our results cannot account for evolutionary responses to cell-to-cell interactions (including the exchange of bacteria that can mediate phytoplankton fitness [64]). However, our results align with other studies in bacteria where evolution with multiple competitors led to maladaptation [11] and slowed adaptation [6]. An important consideration is that we evolved species under constant conditions; environmental fluctuations might be required to evolve niche differentiation [62,65]. Under constant conditions, selection can drive species to convergence towards similar phenotypes. While niche convergence can be an equalizing mechanism that reduces fitness differences [66,67], it also reduces the stabilizing effects that support coexistence [68]. Thus, in stable environments, small differences in competitive ability might over time enable a single dominant species to competitively exclude all others.

Despite differences in community evenness, there was perfect compensation with respect to biovolume at community level. The dominant species almost perfectly counterbalanced the decline in abundance of the competitors, indicating that species loss does not necessarily affect community functioning [69]. The stability of biovolume also shows that coevolution does not always improve community productivity. These conclusions cannot be easily extended to all aspects of productivity. While evolving with competitors did not alter total biovolume, it increased community oxygen fluxes, even if only transiently. It seems that competitive interactions might affect biovolume and oxygen fluxes differently, over both ecological [70] and evolutionary timescales, highlighting the need to measure multiple functions simultaneously [71]. This result accords with the effects of eco-evolutionary dynamics observed in bacteria, albeit in different studies: Castledine *et al.* [11] found no difference in biomass productivity between communities of monoculture- or polyculture-evolved species; while Lawrence *et al.* [4] found that communities of polyculture isolates had significantly higher productivity measured as CO_2_ production rate than communities of monoculture isolates. The differences in energy fluxes we observed in our study were marginal but still the, however little, extra energy produced by polyculture isolates was not used for biomass production. We hypothesize that this ‘extra’ energy could have been used to generate a metabolite or cue by some of the species in the community (e.g. the dominant *Amphidinium*) [72], such as an allelopathic cue or a toxin, that would have been produced only in polyculture (i.e. when evolved with other species) [4,73,74]. However, without analysis of the transcriptome or metabolites, it is not possible to test this hypothesis.

Compensatory dynamics are well documented in ecological communities [75,76] and our results suggest that they might be maintained as species (co)evolve—at least for some functions such as biomass production. While these results should be explored in more complex and diverse systems, communities can display adaptive dynamics that allow for the maintenance of community functioning even as species traits and competitive ability change over time [77]. This stability is encouraging, particularly in light of rapid environmental change [78], but also suggests that constraints on the productivity of communities are not well understood [79,80].

## 5. Conclusion

In summary, our results indicate that evolution between competitors does not majorly alter key aspects of communities, such as species hierarchies and community biomass production, but can still have important consequences on their diversity and functioning. Evolving with competitors seems to reduce coexistence by strengthening competitive differences between species, at least when species compete for essential resources in stable environments. This result is consistent with empirical and theoretical work showing that evolution is unlikely to favour coexistence [53,55]. Importantly, small changes in community assembly and diversity can affect key ecosystem functions such as oxygen production, and possibly their stability— although this remains to be tested. Overall, these results highlight the importance of measuring multiple aspects of community functioning because the effects of multi-species evolution may not be uniform across them.

## Data Availability

All data and code have been deposited in Figshare and can be assessed here [81]. Supplementary material is also available online [82].
